# The Psychological Impact and Behavioral Changes Among the Medical Students of Islamabad and Rawalpindi Due to the COVID-19 Pandemic

**DOI:** 10.7759/cureus.59860

**Published:** 2024-05-08

**Authors:** Mariam Masood, Jaweria Kiani, Seemal Iftikhar, Aamna Faisal

**Affiliations:** 1 Medicine, Shifa International Hospital Islamabad, Islamabad, PAK

**Keywords:** lifestyle, pandemic, psychological impact, behavioral changes, anxiety, covid-19

## Abstract

Introduction

The onset of the COVID-19 pandemic necessitated a shift in global lifestyles as individuals sought to safeguard themselves and their loved ones from the virus. This adaptation involved embracing a distinct way of life marked by social distancing, reduced outdoor engagements, and home confinement. Consequently, this period of quarantine led to diminished social interactions, challenges in accessing essential resources such as food, heightened unemployment rates, and increased burden on healthcare systems. Understandably, these circumstances gave rise to heightened emotions including fear, depression, and anxiety. In response to these dynamics, our research aimed to explore the psychological and behavioral shifts among medical students residing in Islamabad and Rawalpindi (the twin cities of Pakistan) during the year 2020 amidst the COVID-19 pandemic.

Methods

A structured, self-administered questionnaire was constructed, based on previously conducted surveys, assessing the psychological impact and behavioral changes due to the COVID-19 pandemic. The questionnaire was made available online through Google Forms and was provided to students of the various medical colleges of the twin cities of Pakistan. The results were further stratified based on gender.

Results

Categorical data were collected from 400 medical students studying in Rawalpindi and Islamabad. The negative psychological impact was shown by increased stress, 260 (65%), feeling of less energy, 211 (52.8%), and increased anxiety with upper respiratory symptoms, 202 (50.5%). Behavioral changes were also a reflection of the psychological changes depicted by an increased use of disinfectants, 256 (64%), increased desire to clean surfaces, 262 (65.6%), increased use of soaps and detergents, 300 (75%), reduced number of times one left their house 281, (70.3%), and decreased consumption of food products from outside, 226 (56.5%). When compared between the two genders, females had significantly increased stress levels (p=0.034), decreased food consumption from outside (p=0.026), and increased avoidance of people not wearing masks (p=0.001).

Conclusion

Through our study, we identified the various psychological and behavioral changes among our population due to the COVID-19 pandemic. Our study not only highlights these changes but also discusses the various ways to address them. This study would help relevant organizations to understand the broader aspect of how this pandemic has affected individual lives and will also give them ideas regarding how to cater to these changes in a positive way.

## Introduction

Pneumonia caused by severe acute respiratory syndrome coronavirus 2 (SARS-CoV-2) infection emerged in Wuhan City, Hubei Province, China, in December 2019. By February 11, 2020, the World Health Organization (WHO) officially named the disease resulting from infection with SARS-CoV-2 as coronavirus disease 2019 (COVID-19). COVID-19 represented a spectrum of clinical manifestations that typically included fever, dry cough, and fatigue, often with pulmonary involvement. SARS-CoV-2 was highly contagious, and most individuals within the population at large were susceptible to infection [1].

The outbreak was declared by WHO as a global pandemic on March 11, 2020. As of June 17, 2020, 7.9 million people were confirmed cases of the virus globally, and it had taken 435,000 lives. The health impact led to an outbreak of fear and anxiety, which manifested as behavioral changes among different populations. Within a few months of the first report, SARS-CoV-2 had spread across China and worldwide, reaching a pandemic level. As COVID-19 had triggered enormous human casualties and serious economic loss, it posed a global threat [2].

In these trying circumstances, people worldwide had to adopt a unique lifestyle in a bid to protect themselves and their families from the virus. This new lifestyle endorsed social isolation in the form of minimizing outdoor activities and quarantining at home. The quarantine resulted in decreased physical interaction with residents of other homes, increased difficulty in obtaining the basic necessities of life such as food, increased unemployment, and an increased burden on healthcare facilities caused by the virus contraction. In light of these circumstances, it was not surprising that emotions such as fear, depression, and anxiety were on the rise. To define, anxiety is the feeling of fear that occurs when faced with threatening or stressful situations [3]. Our research group aimed to perform this study to recognize the extent to which students’ conduct changed owing to these negative emotions.

## Materials and methods

We carried out a web-based descriptive cross-sectional study in June 2020 in Islamabad and Rawalpindi (the twin cities of Pakistan). Our sample size was calculated through Open Epi calculator, and our estimated sample size was 400. The sampling population comprised all enrolled students in various medical universities of Islamabad and Rawalpindi, including Army Medical College (AMC), Rawalpindi Medical University (RMC), Islamabad Medical and Dental College (IMDC), and Shifa College of Medicine (SCM), and their identities were confirmed by their university ID numbers. We used the non-probability convenient sampling technique. A self-administered questionnaire was constructed by the authors on the basis of previously conducted surveys with regard to Middle East respiratory syndrome [4]. To ensure the validity of the questionnaire, a pilot study comprising 20 participants was conducted. The questionnaire was sent to the participants via Google Forms. The study participants were enrolled students in the aforementioned universities and were between 18 and 25 years of age. Any student who graduated in 2020 or was to begin their university experience in 2020, any student who was on an exchange program, or any student who was above or below the age limits were excluded. The research team made sure that the participants understood the questions and that there was no ambiguity regarding questions by providing their official emails at the end of the consent form. The study was started after ethical clearance from the Institutional Review Board of Shifa Tameer-e-Millat University. All students participating in the study were given detailed information about the purpose of the research and how their information will be used. Written informed consent was taken from each participating individual in the beginning of the questionnaire form. The participants had the choice to withdraw their data from the study at any time if desired. Full anonymity was ensured. The comfort of the participants was our utmost priority. The information provided was not shared with any third party and was solely for research purposes.

The questionnaire consisted of 28 questions. In the first part, the participant’s demographics including name, age, gender, and university names were asked. The second part of the questionnaire was then divided into two parts including psychological and behaviorial impacts of the pandemic. The psychological part investigated various aspects including stress, patience, and anxiety, whereas the behavioral part investigated the various lifestyle changes as a result of the pandemic. Each question had a “Yes,” “No,” or “Maybe” response or an “Increased,” “Decreased,” or “Same as before” response. The data collected were analyzed using SPSS (Statistical Package for Social Sciences) Version 24 (IBM Corp., Armonk, NY). The chi-square test was applied to compare differences based on gender, and a p-value of <0.05 was considered significant.

## Results

Data were collected from 400 medical students studying in Rawalpindi and Islamabad, who had completed the questionnaire consisting of 28 categorical questions. The mean age of our participants was 21.85 (SD: 1.87) years. Of the 400 participants, 240 (60%) were female and 160 (40%) were male. These demographics are listed in Table 1. We divided our results into two broad categories: a) psychological impact of the COVID-19 pandemic on students and b) behavioral changes among students during the COVID-19 pandemic.

**Table 1:** Demographics

Variables	N (%)
Total Participants	400
Male	160 (40%)
Female	240 (60%)
Mean age	21.85 (SD: 1.87)

Psychological impact

The first part of our questionnaire investigated the various psychological impacts of COVID-19 on students, as shown in Table 2 and Table 3. Results showed that 260 (65%) of our participants felt more stressed during the COVID-19 pandemic, while 202 medical students in the twin cities claimed that they became anxious over getting a sore throat, making it a solid (50.5%) of our total sample size, of which 129 (63.8%) were female students and 73 (41.1%) were male students. Also, 211 (52.8%) of respondents reported feelings of less energy. When asked if their motivation to achieve targets decreased, 181 (45.3%) students said yes, of which 112 (61.8%) were female and 69 (38.1%) were male students. Overall, 188 (47%) students became more conscious about their health during the COVID-19, and 172 (43%) students complained of feeling more aggressive; 151 (37.8%) students said they had a decreased ability to concentrate during the COVID-19 pandemic, whereas 139 (34.8%) students had an increased ability to concentrate. When asked about the level of patience, 166 (41.5%) medical students reported no effect, 115 (28.8%) reported decreased patience, and 119 (29.8%) reported an increase in patience.

**Table 2:** Psychological impact of the pandemic

Questions	Yes	No	Maybe
Did you feel more stressed during the COVID pandemic?	260 (65%)	97 (24.3%)	43 (10.8%)
Did your motivation to achieve things decrease?	181 (45.3)	172 (43%)	47 (11.8%)
Did you become extremely anxious with upper respiratory illness symptoms?	202 (50.5%)	133 (33.3%)	65 (16.3%)
Did you become more conscious about your health during the pandemic?	188 (47%)	157 (39.3%)	55 (13.8%)
Did you feel less energetic during the pandemic?	211 (52.8%)	150 (37.5%)	39 (9.8%)
Did you feel more aggressive during the pandemic?	172 (43%)	166 (41.5%)	62 (15.5%)

**Table 3:** Psychological impact of the pandemic

Statements	Increased	Decreased	Same as before
Your ability to concentrate on your studies	139 (34.8%)	151 (37.8%)	110 (27.5%)
Your patience level	119 (29.8%)	115 (28.8%)	166 (41.5%)

Behavioral impact

In regard to the behavioral impact, as shown in Table 4 and Table 5, the majority of students reported increased disinfection in their homes, 256 (64%), along with increased use of cleaning supplies, 300 (75%). It was found that women more often than men reported buying increased amounts of cleaning supplies. Overall, 262 (65.5%) students had an increased desire to clean surfaces at home, of which 154 (58.7%) of affirmative responses belonged to females, 281 (70.3%) students reported they reduced the number of times they left the house, and 73 (18.3%) participants reported no change as compared to before. Also, 256 (64%) of students consciously avoided people who did not adhere to wearing face coverings, of which 171 (66.8%) were female. In terms of eating, 226 (56.5%) students showed a reduction in eating food from restaurants, whereas 125 (31.3%) students continued to eat as before. Time spent sleeping per day was changed, with 225 (56.3%) students reporting increased and 91 (22.8%) students reporting decreased number of sleeping hours per day during the pandemic. There was also an increase in the use of social media. Our results showed that 241 (60.3%) medical students spent more time on the internet. Only 62 (15.5%) said that they spent less time on social media. Purchasing of supplements was not impacted according to our results. In terms of consumption of multivitamins, 187 (46.8%) of students reported increased consumption, whereas 173 (43.3%) reported no change. Furthermore, 196 (49%) students shared that the pandemic did not cause a drop in their grades in medical school, whereas 177 (44.3%) students did see a drop. In terms of appetite, 189 (47.3%) students reported an increase in appetite, 88 (22%) reported a decrease in appetite, 84 (21%) reported no change in appetite. Also, 195 (48.8%) students began to spend more time with their families, with 105 (26.3%) reporting no change. Shopping expenses increased for 123 (30.8%) participants, decreased for 146 (36.5%), and were the same for 131 (32.8%). Interest in current affairs increased for 184 (46%) students, decreased for 90 (22.5%) students, and remained unchanged for 126 (31.5%). Of the students, 153 (38.3%) reported more time spent exercising and 155 (38.8%) reported increased time spent in other hobbies.

**Table 4:** Behavioral impact of the pandemic

Questions	Yes	No	Maybe
Did you have an increased desire to clean surfaces?	262 (65.5%)	91 (22.8%)	47 (11.8%)
Did you start disinfecting surfaces you previously did not?	256 (64%)	87 (21.8%)	57 (14.3%)
Did your daily consumption of Dettol, soap, and sanitizers increase?	300 (75%)	57 (14.3%)	43 (10.8%)
Did you reduce the times you left your house until absolutely necessary?	281 (70.3%)	73 (18.3%)	46 (11.5%)
Did you consciously avoid people not wearing masks?	256 (64%)	78 (19.5%)	66 (16.5%)
Did you start taking vitamin supplements during the COVID-19 pandemic?	187 (46.8%)	173 (43.3%)	40 (10%)
Did you reduce eating from outside?	226 (56.5%)	125 (31.3%)	49 (12.3%)
Did you start hoarding medications?	165 (41.3%)	178 (44.5%)	57 (14.3%)
Did your grades drop during the pandemic?	177 (44.3%)	196 (49%)	27 (6.8%)

**Table 5:** Behavioral impact of the pandemic

Question	Increased	Decreased	Same as before
The number of hours you slept daily	225 (56.3%)	91 (22.8%)	84 (21%)
Your appetite	189 (47.3%)	88 (22%)	123 (30.8%)
Time spent with your families	195 (48.8%)	100 (25%)	105 (26.3%)
Your shopping expenses	123 (30.8%)	146 (36.5%)	131 (32.8%)
Amount of time spent praying	145 (36.3%)	94 (23.5%)	161 (40.3%)
Amount of time you spent exercising	153 (38.3%)	135 (33.8%)	112 (28%)
Amount of time spent on social media	241 (60.3%)	62 (15.5%)	97 (24.3%)
Your interest toward current affairs	184 (46%)	90 (22.5%)	126 (31.5%)
Amount of time spent on hobbies	155 (38.8%)	131 (32.8%)	114 (28.5%)

Comparison between genders

The chi-square test was carried out, and the results for both psychological and behavioral impacts were compared between the two genders, as shown in Tables 6-9. When compared between the two genders (males and females), significant differences were seen in only three domains. Females reported increased stress more often than males. This was statistically significant with a p-value of 0.034, as outlined in Table 6. There were significantly more women who answered affirmatively when asked regarding an increase in avoidance of people not wearing masks compared to men (p-value of 0.001). Significantly, more females reduced eating from outside compared to males (p-value of 0.026), as outlined in Table 8.

**Table 6:** Assessment of the psychological impact of COVID-19 according to gender Values were calculated using the chi-square test; p-values <0.05 were considered statistically significant.

Questions	Male (160)	Yes	No	Maybe	P-value
	Female (240)				
Did you feel more stressed during the COVID pandemic?	M	98 (61.3%)	49 (30.6%)	13 (8.1%)	0.034
	F	162 (67.5%)	48 (20%)	30 (12.5%)	
Did your motivation to achieve things decrease?	M	69 (43.1%)	75 (46.9%)	16 (10%)	0.383
	F	112 (46.7%)	97 (40.4%)	31 (12.9%)	
Did you become extremely anxious with upper respiratory illness symptoms?	M	73 (45.6%)	62 (38.8%)	25 (15.6%)	0.160
	F	129 (53.8%)	71 (29.6%)	40 (16.7%)	
Did you become more conscious about your health during the pandemic?	M	68 (42.5%)	72 (45%)	20 (12.5%)	0.157
	F	120 (50%)	85 (35.4%)	35 (14.6%)	
Did you feel less energetic during the pandemic?	M	82 (51.3%)	62 (38.8%)	16 (10%)	0.899
	F	129 (53.8%)	88 (36.7%)	23 (9.6%)	
Did you feel more aggressive during the pandemic?	M	66 (41.3%)	70 (43.8%)	24 (15%)	0.758
	F	106 (44.2%)	96 (40%)	38 (15.8%)	

**Table 7:** Assessment of the psychological impact of COVID-19 according to gender Values were calculated using the chi-square test; p-values <0.05 were considered statistically significant.

Questions	Male (160)	Increased	Decreased	Same as before	P-value
	Female (240)				
Your ability to concentrate on your studies	M	63 (39.4%)	56 (35%)	41 (25.6%)	0.299
	F	76 (31.7%)	95 (39.6%)	69 (28.8%)	
Your patience	M	41 (25.6%)	54 (33.8%)	65 (40.6%)	0.144
	F	78 (32.5%)	61 (25.4%)	101 (42.1%)	

**Table 8:** Assessment of the behavioral impact of COVID-19 according to gender Values were calculated using the chi-square test; p-values <0.05 were considered statistically significant.

Questions	Male (160)	Yes	No	Maybe	P-value
	Female (240)				
Did you have an increased desire to clean surfaces?	M	108 (67.5%)	36 (22.5%)	16 (10%)	0.647
	F	154 (64.2%)	55 (22.9%)	31 (12.9%)	
Did you start disinfecting surfaces you previously did not?	M	98 (61.3%)	40 (25%)	22 (13.8%)	0.441
	F	158 (65.8%)	47 (19.6%)	35 (14.6%)	
Did your daily consumption of Dettol, soap, and sanitizers increase?	M	116 (72.5%)	30 (18.8%)	14 (8.8%)	0.083
	F	185 (76.7%)	27 (11.3%)	29 (12.1%)	
Did you reduce the times you left your house until absolutely necessary?	M	104 (65%)	36 (22.5%)	20 (12.5%)	0.136
	F	177 (73.8%)	37 (15.4%)	26 (10.8%)	
Did you consciously avoid people not wearing masks?	M	85 (53.1%)	43 (26.9%)	32 (20%)	0.001
	F	171 (71.3%)	35 (14.6%)	34 (14.2%)	
Did you start taking vitamin supplements during the COVID-19 pandemic?	M	71 (44.4%)	78 (48.8%)	11 (6.9%)	0.092
	F	116 (48.3%)	95 (39.6%)	29 (12.1%)	
Did you reduce eating from outside?	M	87 (54.4%)	60 (37.5%)	13 (8.1%)	0.026
	F	139 (57.9%)	65 (27.1%)	36 (15%)	
Did you start hoarding medications?	M	61 (38.1%)	70 (43.8%)	29 (18.1%)	0.182
	F	104 (43.3%)	108 (45%)	28 (11.7%)	
Did your grades drop during the pandemic?	M	71 (44.4%)	79 (49.4%)	10 (6.3%)	0.960
	F	106 (44.2%)	117 (48.8%)	17 (7.1%)	

**Table 9:** Assessment of the behavioral impact of COVID-19 according to gender Values were calculated using the chi-square test; p-values < 0.05 were considered statistically significant

Questions	Male (160)	Increased	Decreased	Same as before	P-value
	Female (240)				
The number of hours you slept daily	M	85 (53.1%)	40 (25%)	35 (21.9%)	0.575
	F	140 (58.3%)	51 (21.3%)	49 (20.4%)	
Your appetite	M	82 (51.3%)	30 (18.8%)	48 (30%)	0.336
	F	107 (44.6%)	58 (24.2%)	75 (31.3%)	
Time spent with your families	M	73 (45.6%)	46 (28.8%)	41 (25.6%)	0.346
	F	122 (50.8%)	54 (22.5%)	64 (26.7%)	
Your shopping expenses	M	49 (30.6%)	61 (38.1%)	50 (31.3%)	0.835
	F	74 (30.8%)	85 (35.4%)	81 (33.8%)	
Amount of time spent praying	M	57 (35.6%)	41 (25.6%)	62 (38.8%)	0.719
	F	88 (36.7%)	53 (22.1%)	99 (41.3%)	
Amount of time you spent exercising	M	55 (34.4%)	58 (36.3%)	47 (29.4%)	0.427
	F	98 (40.8%)	77 (32.1%)	65 (27.1%)	
Amount of time spent on social media	M	97 (60.6%)	24 (15%)	39 (24.4%)	0.978
	F	144 (60%)	38 (15.8%)	58 (24.2%)	
Your interest toward current affairs	M	77 (48.1%)	34 (21.3%)	49 (30.6%)	0.796
	F	107 (44.6%)	56 (23.3%)	77 (32.1%)	

## Discussion

Studies have shown time and time again how public health epidemics can have major psychological and behavioral consequences on individuals, which can be expressed as anxiety, fear, lack of energy and motivation, emotional instability, and hopelessness. College and university-going students are particularly considered a population at risk [5]. The most commonly reported changes in our study were increased levels of stress and anxiety, lack of energy and motivation, increased desire to clean surfaces, increased use of disinfectants, and lesser frequency of going out.

One of the major findings in this study was an increased level of stress, which was reported by 260 (65%) of students. Similar results were reported by a study conducted in Ireland, which investigated different stressors and levels of stress in medical students due to COVID-19. It reported that 54.5% of respondents had stress levels ranging from moderate to extreme. Significant stressors included changes in the education system, such as transitioning to online classes and assessments, decreased social interaction, international student status, and fear and worries about one's own health and the health of loved ones [6].

A study conducted in Saudi Arabia compared pre-pandemic stress levels of medical students to perceived stress levels during the pandemic and found that stress levels during the pandemic (20.6) compared to pre-pandemic (11.6) were significantly higher (p=0.001) [7]. A study conducted on medical students in Pakistan during the pre-pandemic period revealed stress in 42% of the students, while the remaining 58% showed no stress [8]. In our study, we had a higher percentage of students who were found to be stressed. This could be a result of additional stressors on students due to the adaptation of the new online system for teaching and the continuously prevailing fear of one's health. When compared between the two genders, the result for stress level was significantly more in females. Anxiety disorders are among the most prevalent mental disorders, and women are at much higher risk of developing any anxiety disorder [9].

One interesting finding in our study was that 151 (37.8%) students reported a decreased ability to concentrate during the COVID-19 pandemic as opposed to 139 (34.8%) students who claimed to have had an increased ability to concentrate. This could be due to an increased amount of time spent at home to indulge in other distractions such as watching television, spending time on social media, and sleeping. Students who are easily distracted find it difficult to concentrate on academics at home [10]; 181 (45.3%) students also reported a decrease in their motivation to achieve targets, which might be due to the uncertainty of health and life as a result of the ongoing pandemic.

It was reported in a number of studies that people became more mindful of their health because of the pandemic. We had similar findings; 188 (47%) respondents claimed to have become more health conscious, and 187 (46.8%) respondents reported the use of multivitamins that were not prescribed by doctors. A study conducted on students in Serbia also reported a significant increase in the use of dietary supplements not prescribed by physicians (40.2 %) during the pandemic compared to pre-pandemic (33.7%, p = 0.008) [11]. While increased consciousness about one’s health was a positive finding as it showed increased awareness regarding the importance of good health, an increased use of non-prescribed medications including supplements was a negative impact that came along with increased health consciousness. Specifically, it has been established that COVID-19 impacted the daily dietary habits of adults [12]. Multiple studies revealed an increased consumption of healthy food markers such as fruits, vegetables, and legumes during the pandemic [13,14]. Another study conducted on Greek and Swedish students during the COVID-19 pandemic revealed that the proportion of ultraprocessed food in the Greek and Swedish students’ main meals decreased during the COVID-19 pandemic versus before the pandemic, while the proportion of main meals with vegetables and/or fruits increased [15]. Similarly, in our study, we found out that a large portion of our study population, 226 (56.5%), reduced eating from outside and preferred home-cooked food. This might be because of an increased fear of contamination of food, probably due to lack of awareness early on regarding transmission.

Numerous studies have been conducted on examining changes in hygiene habits due to COVID-19. The most common ones were wearing masks, hand hygiene, markedly increased use of disinfectants, and frequent use of hand sanitizers [16]. The hand washing frequency rapidly increased as WHO declared the COVID-19 pandemic, May 2020, with an increasing mean hand washing frequency of four times before the pandemic to eight times during the pandemic [17]. It is safe to say that awareness programs regarding the importance of hygiene habits have played a significant role in decreasing the disease burden of not only COVID-19 but also other droplet infections. Our study also reported similar results as 262 (65.5 %) of participants claimed that they had an increased desire to clean surfaces and 300 (75%) reported increased use of disinfectants.

Other major lifestyle changes that were established in numerous studies included increased sleep duration [18], increased appetite and frequency of meals [11], decreased physical activity, particularly walking [19], increased time spent on electronic gadgets [20], and decreased frequency of leaving home. Compared to the pre-lockdown period, there was a shift to a later bedtime and waking time, with a reduction in night-time sleep and an increase in daytime napping [20]. However, our study results reported an overall increase in sleeping time in 225 (56.3%) of the participants. Our study also reported that 281 (70.3%) of students reduced the number of times they left their homes, and 241 (60.3%) reported that they spent more time on social media.

COVID-19 has undeniably emerged as the most devastating health emergency in recent times [21]. Medical students are considered a vulnerable population when it comes to mental health and stress because of the demanding academics and brutal competition [22]. Keeping in mind the large percentage of students who are implicated by the psychological and behavioral effects of the pandemic [23,24], steps should be taken to find solutions to these problems. Considering the trend shown in our research, a number of steps can be taken to counter these impacts on students. We believe that universities and colleges should be well-versed in addressing the mental health needs of students. New counselling programs and support groups with professionals should be introduced to students. Students should be taught about adaptability and meditation. Risk stratification tools should be used to identify students with clinical levels of anxiety and depression so that they can get timely help. Efforts should be made to change the common mindset of our people regarding help-seeking for one's mental health. To cater to the negative behavioral changes such as the increased use of non-prescribed medications, it is absolutely essential for the government to take steps to make focused groups and run awareness campaigns to clear any misconceptions regarding the spread of the virus and infectivity so that people can make rational decisions and avoid unnecessary behaviors that might seem important to them for their health protection.

The strength of our study is that we have thoroughly studied the behavioral impact of COVID-19 on medical students of the twin cities of Pakistan, adding more to the evidence pool already present. We believe that more studies should be conducted on further identifying specific stressors and coping strategies. The weak point of our study is that we used online questionnaires for our data collection, which are subject to interpretation bias. Although our data were strictly confidential, we still believe that since we investigated topics such as stress and anxiety, there is a possibility that some students may have given the socially desirable answer. Despite the fact that various aspects of anxiety symptoms and psychological impacts were investigated in our study, there is still a need for more studies to further thoroughly investigate these psychological impacts. Another limitation can be the small sample size and non-random sampling of the sample, which may limit the generalization of this study.

## Conclusions

Through our study, we identified the various psychological and behavioral changes among our population due to the COVID-19 pandemic depicted by increased levels of stress, anxiety, and certain changes in behavior. Our study not only highlighted these changes but also discussed the various ways to address them. This study would help relevant organizations to understand the broader aspect of how this pandemic has affected individual lives, especially students, and will also give them ideas regarding how to cater to these changes in a positive way. Moreover, large-scale studies should be continued for further understanding these impacts and the need for further targeted solution programs if required in the future.
